# Extracorporeal Carbon Dioxide Removal in Acute Respiratory Distress Syndrome: Physiologic Rationale and Phenotype-Based Perspectives

**DOI:** 10.3390/medicina62020236

**Published:** 2026-01-23

**Authors:** Raffaele Merola, Denise Battaglini, Silvia De Rosa

**Affiliations:** 1 Anesthesia and Intensive Care Medicine, Department of Neurosciences, Reproductive and Odontostomatological Sciences, University of Naples Federico II, 80131 Naples, Italy; 2 Department of Surgical Sciences and Integrated Diagnostics (DISC), University of Genoa, 16132 Genova, Italy; 3 Anesthesia and Intensive Care, IRCCS Azienda Ospedaliera Metropolitana, 16132 Genova, Italy; 4 Centre for Medical Sciences, University of Trento, 38122 Trento, Italy; 5 Anesthesia and Intensive Care, Santa Chiara Hospital, Azienda Provinciale per i Servizi Sanitari di Trento, 38122 Trento, Italy

**Keywords:** acute respiratory distress syndrome (ARDS), extracorporeal carbon dioxide removal (ECCO_2_R), mechanical power, precision medicine, ventilator-induced lung injury (VILI)

## Abstract

Acute respiratory distress syndrome (ARDS) is a major cause of morbidity and mortality despite decades of progress in ventilatory support. Mechanical ventilation, while essential for oxygenation, may exacerbate lung injury through excessive mechanical power delivery, even when using lung-protective strategies. Extracorporeal carbon dioxide removal (ECCO_2_R) was conceived to enable “ultra-protective” ventilation, allowing for further reductions in tidal volume and respiratory rate by selectively removing CO_2_ at low extracorporeal blood flows, typically between 0.3 and 1.0 L/min. This physiological decoupling of ventilation and gas exchange aims to mitigate ventilator-induced lung injury (VILI) while maintaining adequate acid–base homeostasis. Although early physiological studies demonstrated feasibility, large, randomized trials have failed to show a survival benefit and have raised concerns about bleeding and technical complications. Recent evidence suggests that these neutral outcomes may stem from the biological and physiological heterogeneity of ARDS rather than from inefficacy of the intervention itself. Patients with high driving pressures, poor compliance, or hyperinflammatory phenotypes may derive greater benefit from ECCO_2_R-mediated mechanical unloading. Ongoing technological improvements, including circuit miniaturization, enhanced biocompatibility, and integration with renal replacement therapy, have improved safety and feasibility, yet the procedure remains complex and resource-intensive. Future research should focus on phenotype-enriched trials and the integration of ECCO_2_R into precision ventilation frameworks. Ultimately, ECCO_2_R should be regarded not as a universal therapy for ARDS but as a targeted physiological tool for selected patients in experienced centers.

## 1. Introduction

The acute respiratory distress syndrome (ARDS) is one of the most challenging conditions encountered in intensive care medicine [1]. It is defined by non-cardiogenic pulmonary edema, profound hypoxemia, and bilateral infiltrates, ARDS represents a final common pathway of diverse insults leading to diffuse alveolar damage [2]. Despite decades of progress in supportive care, mortality remains substantial, typically ranging between 30% and 45% [3,4]. Mechanical ventilation is the cornerstone of management, yet even lung-protective strategies—low tidal volumes and limitation of plateau pressures—can perpetuate or exacerbate ventilator-induced lung injury (VILI). This ongoing risk underscores the delicate balance clinicians must achieve between providing sufficient respiratory support and minimizing iatrogenic lung damage, which continues to limit improvements in patient outcomes [5,6].

Extracorporeal carbon dioxide removal (ECCO_2_R) was developed to address this limitation by selectively removing CO_2_ through an extracorporeal circuit operating at blood flow rates markedly lower than those required for extracorporeal membrane oxygenation (ECMO) [7,8]. By reducing the patient’s CO_2_ burden, ECCO_2_R permits further reductions in alveolar ventilation, tidal volume, and respiratory rate—a concept known as “ultra-protective ventilation”. This approach aims to minimize mechanical energy delivery to the lung and thereby mitigate VILI [9].

However, translating this appealing physiological concept into clinical benefit has proved difficult. Early physiological studies demonstrated safety and feasibility, but large randomized controlled trials (RCTs) failed to show a mortality advantage and raised concerns regarding complications, particularly bleeding [9,10,11]. These mixed results underscore that the success of ECCO_2_R may depend on precise patient selection, timing, and integration into individualized ventilatory strategies.

Additionally, the recognition of ARDS as a heterogeneous syndrome encompassing distinct biological and physiological sub-phenotypes has revitalized interest in individualized approaches [12,13,14]. This heterogeneity explains why uniform therapies often produce neutral clinical results: the mechanical burden of ventilation and the inflammatory response to lung stress vary markedly between patients [12,13,14]. Within this framework, ECCO_2_R should not be regarded as a treatment for ARDS but as a precision tool targeting specific patient subsets who may derive disproportionate benefit. The aim of this narrative review is to examine the physiological basis, clinical experience, and potential for individualized, phenotype-driven application of ECCO_2_R in ARDS management.

## 2. Search Strategy

This narrative review was informed by a focused, non-systematic literature search aimed at identifying key physiological, clinical, and translational studies on ECCO_2_R in ARDS.

The electronic databases PubMed, MEDLINE and Scopus were searched for articles published up to November 2025. The search strategy combined relevant keywords and Medical Subject Headings (MeSH), including “extracorporeal carbon dioxide removal”, “ECCO_2_R”, “acute respiratory distress syndrome”, “ARDS”, “mechanical power”, “ultra-protective ventilation”, and “phenotypes”.

Additional relevant studies were identified through manual screening of reference lists from key reviews and seminal articles. Priority was given to physiological studies, randomized controlled trials, high-quality observational studies, consensus documents, and recent reviews published in peer-reviewed journals.

Given the narrative nature of this review, no formal systematic selection process or meta-analytic approach was applied.

## 3. Physiological Rationale for ECCO_2_R

### 3.1. Gas Exchange Physics

The physiological mechanisms underlying CO_2_ and O_2_ transport differ fundamentally. Oxygenation is primarily constrained by hemoglobin saturation and consequently necessitates high extracorporeal blood flows, often several liters per minute, to achieve clinically meaningful gas exchange. In contrast, CO_2_ is predominantly carried in dissolved and bicarbonate forms and equilibrates rapidly across the alveolar–capillary membrane.

This allows CO_2_ clearance to be achieved with much lower extracorporeal blood flow rates—typically between 0.3 and 1.0 L/min, representing 10–20% of cardiac output.

Thus, under optimized conditions—including adequate membrane surface area, high sweep-gas flow, and favorable pre-membrane CO_2_ tension—ECCO_2_R can theoretically remove 150–200 mL/min of CO_2_, approximating total metabolic production in many adult patients [8,15,16,17]. This reflects the physiological basis for employing ECCO_2_R: by partially or entirely removing a patient’s CO_2_ production, the burden of CO_2_ elimination via the lungs is correspondingly reduced. Consequently, ECCO_2_R may be utilized to attenuate the intensity of mechanical ventilation, thereby mitigating VILI, and/or to reduce the patient’s work of breathing, even in instances where the CO_2_ removed does not encompass the entirety of endogenous CO_2_ production [15].

The key determinant of CO_2_ removal is the product of blood flow and the difference in CO_2_ content across the membrane, modulated by sweep gas flow and membrane surface area [18]. CO_2_ removal exhibits a hyperbolic relationship with extracorporeal blood flow; increases in flow initially yield steep gains in clearance, which progressively plateau once most dissolved CO_2_ and bicarbonate-derived CO_2_ are eliminated [19,20,21]. This nonlinearity has critical implications for device efficiency and patient management, as excessive flow increases may offer minimal additional clearance but magnify risks of hemolysis or access complications [22,23].

From a systems perspective, the introduction of ECCO_2_R effectively creates two parallel routes for CO_2_ elimination: the native lung and the artificial “membrane lung.” The total CO_2_ clearance thus depends on the balance between extracorporeal and alveolar contributions [8,21]. By removing a significant portion of the metabolic CO_2_ load extracorporeally, the demand for alveolar ventilation decreases, permitting substantial reductions in tidal volume and respiratory rate while maintaining arterial CO_2_ and pH within physiological range values [9,24]. The extent of feasible ventilatory downscaling depends on baseline CO_2_ production, circuit efficiency, and systemic metabolic demand [25].

A crucial physiological aspect of ECCO_2_R is its impact on acid–base homeostasis [26]. Extracorporeal removal of CO_2_ shifts the equilibrium of the bicarbonate–carbonic acid buffer system:

CO_2_ + H_2_O ↔ H_2_CO_3_ ↔ H^+^ + HCO_3_^−^

By selectively removing CO_2_ from the bloodstream, ECCO_2_R shifts this equilibrium to the left, decreasing hydrogen ion concentration and consequently increasing blood pH. When renal function remains intact, compensatory adjustments in bicarbonate reabsorption and hydrogen ion excretion gradually restore equilibrium, limiting excessive alkalinization [27].

This interplay explains why ECCO_2_R can normalize or even alkalinize blood pH despite markedly reduced alveolar ventilation—effectively decoupling ventilation from acid–base regulation. Thus, any decrease in PaCO_2_ via extracorporeal clearance elevates pH unless balanced by a proportional decline in plasma bicarbonate concentration. However, excessive or abrupt CO_2_ removal may outpace the kidney’s compensatory capacity, producing metabolic disequilibrium and systemic alkalosis [26,27]. In practice, this necessitates a physiologically guided titration of sweep gas flow, balancing CO_2_ clearance with the body’s intrinsic buffering and renal adaptive mechanisms.

### 3.2. Native and Artificial Lung Interaction

The concept of lung protection has evolved from static pressure limitation to a dynamic understanding of “mechanical power”—the rate at which energy is transferred to the respiratory system, representing the intensity of ventilation [6]. Mechanical power integrates the effects of tidal volume, driving pressure (ΔP), respiratory rate, flow pattern, and resistance [28]. Even within conventionally “safe” tidal volumes (6–8 mL/kg predicted body weight [PBW]), high respiratory rate or low compliance can produce excessive mechanical power and consequent injury [29,30,31].

ECCO_2_R modifies this equation by allowing deeper reductions in tidal volume (to 3–4 mL/kg) and respiratory rate (often <20 breaths/min) while maintaining acceptable gas exchange [32]. This strategy, known as ultra-protective ventilation, can reduce mechanical power by 30–50% compared with standard protective settings [24]. The physiological benefit derives not only from lower static pressures but also from fewer cyclic deformations and reduced regional strain heterogeneity [26]. Experimental and clinical studies demonstrate that ultra-protective ventilation supported by ECCO_2_R attenuates alveolar inflammation, reduces cytokine release, and mitigates histologic lung injury compared to standard protective ventilation [33,34,35]. The benefits derive not only from lower peak and plateau pressures but also from decreased cumulative mechanical energy exposure over time.

**Figure 1:**
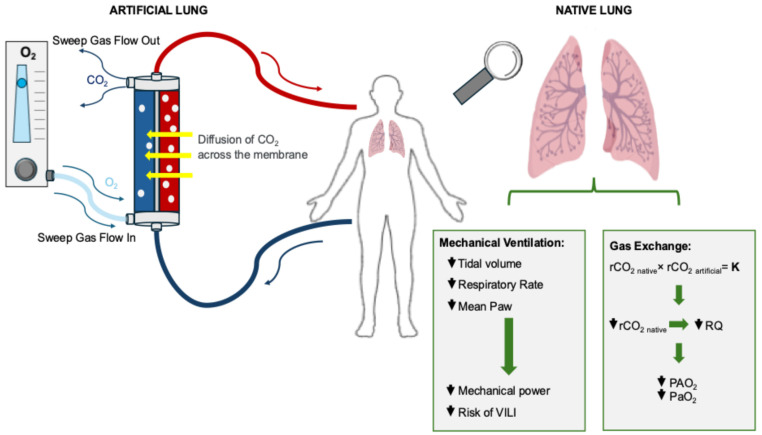
Interaction between artificial and native lung. CO_2_ diffuses across the artificial lung membrane and is removed by the sweep gas, while oxygen transfer remains negligible. As extracorporeal CO_2_ removal increases, native lung CO_2_ elimination decreases, resulting in a lower overall respiratory quotient (RQ = VCO_2_/VO_2_). According to the alveolar gas equation, this reduction in RQ decreases alveolar and arterial oxygen tension (PAO_2_ and PaO_2_) even if FiO_2_ is unchanged. The reduction in mechanical ventilation intensity enabled by extracorporeal CO_2_ removal decreases mechanical power and thereby mitigates the risk of ventilator-induced lung injury (VILI). Abbreviations: K = constant representing the total CO_2_ removal balance; PaO_2_—Arterial Partial Pressure of Oxygen; PAO_2_ = alveolar partial pressure of O_2_; rCO_2 native_ = rate of CO_2_ removed by the native lung; rCO_2 artificial_ = rate of CO_2_ removed by the artificial lung; RQ = Respiratory quotient.

However, these effects are not purely mechanical. Lowering respiratory rate and tidal volume modifies the dynamic interplay between alveolar and extracorporeal gas exchange [21]. During ECCO_2_R, oxygen exchange in the native lung remains largely unchanged, since the amount of oxygen added extracorporeally is minimal. In contrast, the removal of CO_2_ by the native lung decreases in proportion to the CO_2_ eliminated through the extracorporeal support, resulting in a lower respiratory quotient (RQ = VCO_2_/VO_2_). Because alveolar partial pressure of oxygen (PAO_2_) is influenced by both FiO_2_ and the PaCO_2_/RQ ratio according to the alveolar gas equation, a reduction in RQ can lead to a decrease in alveolar and arterial pressure of oxygen even when FiO_2_ is constant (Figure 1) [36,37].

Moreover, during apnea, the composition of alveolar gas is shaped by the nitrogen content in the artificial lung, as alveolar nitrogen equilibrates with it. If the nitrogen concentration in the artificial lung is lower than in the native lung—that is, if the oxygen fraction delivered via the membrane lung exceeds FiO_2_—progressive nitrogen depletion occurs in the native lung [36]. This can promote reabsorption atelectasis in regions with low ventilation/perfusion ratios, thereby increasing the instability of pulmonary units [38]. While this phenomenon has been experimentally observed during apnea, it may also occur regionally under clinical conditions, highlighting the physiological interplay between extracorporeal support and native lung function.

It should be emphasized that nitrogen washout and denitrogenation atelectasis are not directly dependent on extracorporeal blood flow per se, but rather on the fraction of oxygen delivered to the alveoli. In theory, whenever the oxygen fraction delivered by the membrane lung exceeds the ventilator FiO_2_, a net nitrogen washout from the native lung may occur, potentially leading to progressive alveolar denitrogenation and collapse. This phenomenon reflects a physiological redistribution of gas exchange between the artificial and native lung rather than a flow-dependent complication of ECCO_2_R itself.

However, in the context of low-flow ECCO_2_R (0.3–1.0 L/min), extracorporeal oxygen transfer is generally negligible, and the clinical relevance of denitrogenation atelectasis in this setting remains largely theoretical and incompletely quantified. At present, no robust clinical evidence suggests that this mechanism represents a major complication of low-flow ECCO_2_R when compared with better-established risks, such as bleeding or vascular access–related events.

From a physiological standpoint, the greatest potential benefit of ECCO_2_R may arise in patients in whom mechanical power cannot be reduced further by conventional means—those with markedly reduced compliance, high ΔP, or vigorous spontaneous efforts. In such contexts, ECCO_2_R acts as a physiological “decoupler,” allowing safe reduction in ventilatory intensity without compromising systemic CO_2_ homeostasis.

### 3.3. Systemic and Respiratory Drive Effects

CO_2_ is not only a waste product but also a potent regulator of respiratory drive and acid–base balance. Hypercapnia triggers increased ventilatory effort through central chemoreceptor activation, and in spontaneously breathing or partially supported patients, this can translate into excessive inspiratory effort, high transpulmonary pressures, and patient self-inflicted lung injury (P-SILI) [6,39,40].

Beyond enabling ultra-protective ventilation, ECCO_2_R may attenuate excessive respiratory drive and effort in spontaneously breathing patients, reducing transpulmonary pressure swings and the risk of P-SILI. This dimension is particularly relevant in modern ARDS management, where spontaneous ventilation is increasingly common. The ability to modulate respiratory drive positions ECCO_2_R as a potential adjunct to prevent the deleterious loop between hypercapnia, vigorous effort, and lung injury in spontaneously breathing ARDS patients [41,42,43].

Conversely, excessive CO_2_ removal can suppress respiratory drive, leading to hypoventilation, atelectasis, or alkalosis. The optimal target, therefore, is not normocapnia but controlled permissive hypercapnia that balances ventilatory comfort, pH stability, and lung protection [44]. Individualized adjustment of CO_2_ clearance—guided by arterial blood gases, ventilatory effort monitoring, and clinical response—is essential.

Systemically, the modulation of PaCO_2_ exerts profound effects on both hemodynamics and cerebral blood flow, reflecting its central role in physiological homeostasis. Moderate hypercapnia can enhance tissue oxygen delivery through multiple interconnected mechanisms: it shifts the oxyhemoglobin dissociation curve to the right, facilitating oxygen unloading at the tissue level, and simultaneously increases cardiac output by stimulating sympathetic activity [45,46,47]. In contrast, abrupt or excessive hypocapnia can trigger pronounced vasoconstriction, particularly in the cerebral circulation, leading to reduced perfusion and potentially compromising oxygen delivery despite adequate arterial oxygen content [48,49,50]. Thus, the rate and extent of CO_2_ removal must be individualized, particularly in patients with intracranial pathology or circulatory instability.

The extracorporeal circuit itself introduces additional physiological perturbations, including hemodilution, mild inflammation, and exposure of blood to artificial surfaces. Minimizing these effects requires high-quality biocompatible materials, optimized anticoagulation, and experienced multidisciplinary oversight [8,51].

Together, these considerations highlight that the physiological impact of ECCO_2_R extends beyond simple CO_2_ clearance—it reshapes the entire homeostatic system governing ventilation, circulation, and metabolism. Translating this physiological promise into clinical benefit has proven challenging, largely due to the heterogeneity of ARDS and the limited ability of current trials to identify patients in whom mechanical unloading is truly meaningful [12,13,14].

## 4. Evidence and Clinical Experience

The concept of partial ECCO_2_R was introduced almost fifty years ago by Gattinoni and colleagues, who combined extracorporeal decarboxylation with low-frequency ventilation to mitigate VILI in patients with severe ARDS [15,16,36,37,52]. However, early clinical implementation faced major technical and physiological limitations. The devices available at the time were characterized by high membrane resistance and limited gas-exchange efficiency, resulting in suboptimal CO_2_ clearance. In addition, the use of large-bore cannulas and non-optimized anticoagulation protocols led to a high incidence of complications, including bleeding, hemolysis, and circuit thrombosis. These challenges substantially limited the dissemination of ECCO_2_R and hindered its clinical translation for decades. Subsequent advances in membrane technology and extracorporeal circuit design have, however, renewed interest in its potential as an adjunct to lung-protective ventilation, prompting a series of feasibility and safety investigations and RCTs (Table 1) [7].

ECCO_2_R enables the ultra-protective targets described above, allowing further reductions in tidal volume and respiratory rate beyond conventional lung-protective ventilation [33]. Bein et al. conducted a randomized trial comparing ultraprotective ventilation (3 mL/kg PBW) enabled by arteriovenous ECCO_2_R (AV-ECCO_2_R) with standard protective ventilation [53]. Although no significant difference was observed in ventilator-free days at day 28 due to limited sample size, post hoc analysis revealed shorter mechanical ventilation duration in patients with severe hypoxemia (PaO_2_/FiO_2_ < 150 mmHg) treated with ECCO_2_R. Beyond enabling ultraprotective ventilation, ECCO_2_R may also facilitate conventional lung-protective strategies (6 mL/kg PBW). For example, Moss et al. observed a sustained reduction in peak inspiratory pressures following ECCO_2_R initiation in nine ARDS patients [54]. A systematic review of 14 studies including 495 patients confirmed that ECCO_2_R is feasible, promotes lower tidal volume ventilation, and is associated with increased ventilator-free days, although survival benefit was not demonstrated [55].

**Table 1:** Main prospective studies reporting on ECCO_2_R for Acute Respiratory Distress Syndrome.

Study (Year)	Study Design	No. of Patients	Device Used	Membrane Surface Area (m^2^)	Blood Flow (mL/min)	Duration of ECCO_2_R	Tidal Volume on ECCO_2_R (mL/kg)	Objectives/Outcomes	Key Findings
Terragni et al. (2009) [33]	Physiological, single-center	32	VV Decap^®^, Hemodec	0.33	191–422	72 h	4.2 ± 0.3	Pulmonary cytokines and inflammatory biomarkers	Reduction in pulmonary cytokines and inflammatory biomarkers
Bein et al. (XTRAVENT, 2013) [53]	Randomized controlled trial	79	iLA AV shunt, Novalung	1.3	1300 ± 200	7.4 ± 4 days	3	Ventilator-Free Days at day 28	Fewer ventilator-free days in ECCO_2_R group
Fanelli et al. (2016) [56]	Feasibility and safety	15	Hemolung, Alung	0.59	435 ± 60	24 h	4	Correct pH and PaCO_2_	Ph and PaCO_2_ corrected
Schmidt et al. (2018) [57]	Multicenter feasibility and safety	20	VV Prismalung, Baxter	0.32	421 ± 40	24 h	3.98 ± 0.18	Safety and feasibility of a low-flow ECCO R	Confirmed safety and feasibility
Combes et al. (SUPERNOVA Pilot, 2019) [9]	Prospective multicenter, international, phase II	95	Low flow: Hemolung, Alung; High flow: iLA activve, Xenios; CardioHelp, Getinge	0.59 (Hemolung) 1.30 (iLA activve, CardioHelp)	Low flow: 440 [430–480]; High flow: 960 [800–1000]	5 [3–8] days	4	No. of patients who successfully achieved ultra-protective ventilation	78% of patients successfully achieved ultra-protective ventilation
McNamee et al. (REST trial, 2021) [11]	Randomized controlled trial	405	Hemolung, Alung	0.59	350–450	4 ± 2 days	4.5 [4.3–4.8]	Mortality at day 90	Trial stopped because of futility. No mortality benefit at day 90

Subsequent feasibility and safety studies further supported the role of ECCO_2_R in enabling ultraprotective ventilation. In a prospective observational study, Fanelli et al. (2016) demonstrated that ECCO_2_R in moderate ARDS allowed a stepwise reduction in tidal volume to 4 mL/kg while maintaining adequate gas exchange [56]. Similarly, Schmidt et al. integrated low-flow ECCO_2_R systems into continuous renal replacement therapy (CRRT) platforms in patients with mild-to-moderate ARDS, simplifying extracorporeal management and underscoring the potential of multi-organ support systems to enhance lung protection [57]. Collectively, these studies reinforced the feasibility and safety of ECCO_2_R, paving the way for subsequent RCTs.

The SUPERNOVA pilot trial evaluated the feasibility and safety of three ECCO_2_R devices in 95 ARDS patients. ECCO_2_R enabled tidal volume reduction from approximately 6 to 4 mL/kg PBW and significantly decreased ΔP (from 13 to 9 cmH_2_O), with minimal change in PaCO_2_. Serious adverse events were infrequent [9].

The REST trial, a multicenter study enrolling 412 adults with acute hypoxemic respiratory failure (PaO_2_/FiO_2_ < 150 mmHg), randomized patients to receive ultraprotective ventilation facilitated by ECCO_2_R or conventional low tidal volume ventilation [11]. The trial was terminated early for futility and feasibility. Ninety-day mortality did not differ between groups (ECCO_2_R vs. standard care: 41.5% vs. 39.5%; *p* = 0.68), and patients in the ECCO_2_R arm had fewer ventilator-free days. Serious adverse events were more frequent in the ECCO_2_R group, primarily related to bleeding, including intracranial hemorrhage. Several methodological limitations have been highlighted: only 60% of patients met ARDS criteria at randomization, ΔP was <15 cmH_2_O in half of the cohort, and despite severe hypoxemia (median PaO_2_/FiO_2_ 118 mmHg), median PEEP was relatively low (10 cmH_2_O), with only 11% of patients receiving prone positioning. Two days after randomization, reductions in tidal volume (6.3→4.5 mL/kg) and ΔP (15→12 cmH_2_O) were modest, whereas respiratory rate and PaCO_2_ increased (24→27 breaths/min; 54→61 mmHg). These findings suggest that the device used may have provided insufficient CO_2_ clearance to enable full ultraprotective ventilation while controlling respiratory acidosis. Furthermore, most participating centers were inexperienced with ECCO_2_R prior to the trial, which may have contributed to the observed outcomes.

In a prespecified secondary analysis of the REST trial, Boyle and colleagues sought to explore the biological heterogeneity underlying the variable response to ECCO_2_R-facilitated ultra-protective ventilation. The study found no significant reduction in systemic inflammation, as reflected by plasma C-reactive protein concentrations at 72 h, in patients randomized to ECCO_2_R compared with standard care. Interestingly, treatment was associated with higher plasma interleukin-18 (IL-18) concentrations, suggesting possible activation of inflammasome pathways rather than suppression of inflammatory responses. Importantly, there was no evidence of increased hemolysis, as assessed by circulating free hemoglobin. Stratified analyses revealed that patients with elevated baseline IL-18 or a hyperinflammatory subphenotype appeared to experience greater benefit from ECCO_2_R, with trends toward more ventilator-free days and improved 90-day survival, whereas no such advantage—and even potential harm—was observed in non-hyperinflammatory subgroups [58]. These findings provide a plausible biological rationale for the neutral results of the primary REST trial, suggesting that the effects of ECCO_2_R are unlikely to be uniform across the heterogeneous ARDS population. When the physiological premise of ECCO_2_R is aligned with patient-specific biology and lung mechanics, the intervention becomes far more coherent. In unselected populations, however, any potential benefit is diluted by patients who are unlikely to derive meaningful mechanical unloading. Taken together, these data highlight the need for precision-based strategies that integrate biomarker profiling, inflammatory phenotyping and respiratory mechanics data to identify the subgroups most likely to benefit from extracorporeal CO_2_ removal—while ensuring that any mechanical advantages are balanced against its systemic and inflammatory consequences.

## 5. ARDS Subphenotypes and the Precision Application of ECCO_2_R

As previously discussed, ARDS exhibits marked biological and physiological heterogeneity, which likely explains the neutral results of uniform therapeutic strategies. The recognition of such heterogeneity reinforces the rationale for a precision-based approach to interventions such as ECCO_2_R [59,60,61].

Within this framework, patients exhibiting higher ΔP (>15 cmH_2_O) and low respiratory system compliance experience excessive mechanical stress even under conventionally protective ventilation. In these patients, the physiological burden of ventilation often translates into a high mechanical power and an increased risk of VILI [62,63]. ECCO_2_R, by enabling further reductions in tidal volume, respiratory rate and, consequently, minute ventilation, offers the potential to lower both ΔP and mechanical power, thereby attenuating ongoing biophysical injury. Post hoc analyses of previous ECCO_2_R trials support this concept, suggesting that patients with the highest baseline mechanical load may derive the most meaningful physiological benefit from an ultra-protective ventilation strategy facilitated by extracorporeal CO_2_ removal [53].

Another dimension of heterogeneity lies in the biological response to injury. The hyperinflammatory ARDS subphenotype, typified by elevated systemic cytokine levels, endothelial activation, and multi-organ dysfunction, exemplifies a subgroup in which inflammation itself becomes a dominant driver of pathology [64,65,66,67,68]. In this context, mechanical stress from ventilation can amplify the inflammatory cascade through cytokine release, alveolar-capillary barrier disruption, and propagation of systemic injury. By decreasing mechanical power and stress, ECCO_2_R could theoretically attenuate this deleterious interplay between mechanical and biological injury mechanisms, potentially fostering a more favorable inflammatory milieu. However, it must be acknowledged that patients within this subphenotype frequently present with coagulopathy or thrombocytopenia, thereby facing an elevated risk of hemorrhagic complications during extracorporeal support [64,65,66,67,68]. Reconciling this paradox requires a nuanced, phenotype-aware risk–benefit assessment rather than a binary treatment decision. Consequently, the hyperinflammatory phenotype should not be viewed as an absolute indication for ECCO_2_R, but rather as a subgroup in which the potential physiological benefit of mechanical unloading must be weighed against an intrinsically higher hemorrhagic risk. Future trials specifically enriched for hyperinflammatory patients should incorporate adaptive anticoagulation protocols and bleeding-risk stratification to determine whether this paradox can be safely navigated in clinical practice.

Morphological patterns of lung injury further influence the potential response to ECCO_2_R [69,70,71]. Patients with diffuse, non-focal ARDS, in whom strain and stress are globally distributed and recruitment potential is limited, may benefit more from ventilation strategies facilitated by extracorporeal CO_2_ removal. Conversely, in focal ARDS, where atelectatic regions coexist with relatively preserved areas, recruitment maneuvers and prone positioning tend to yield more pronounced physiological improvement, and excessive reduction in ventilatory support may instead promote derecruitment and hypoxemia.

**Figure 2:**
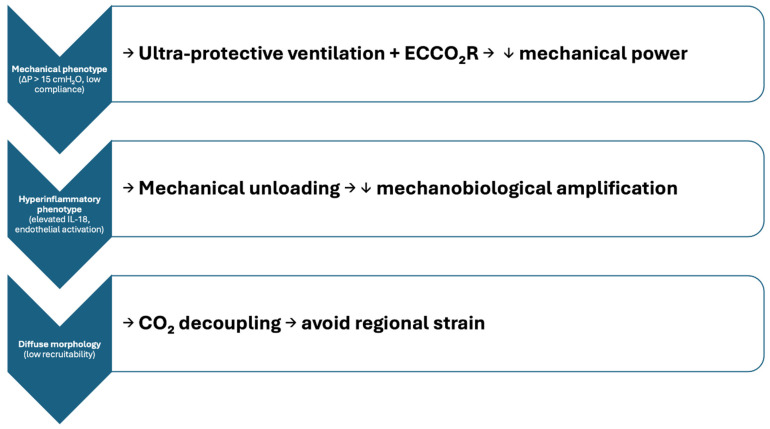
Phenotype-driven rationale for ultra-protective ventilation supported by ECCO_2_R. In patients with a predominantly mechanical phenotype (high ΔP, low compliance), ECCO_2_R enables ultra-protective ventilation by allowing extreme reductions in tidal volume and driving pressure, resulting in lower mechanical power. In hyperinflammatory phenotypes (elevated IL-18 and endothelial activation), mechanical unloading reduces mechanobiological amplification. In diffuse, low-recruitability morphology, CO_2_ decoupling prevents regional strain by avoiding excessive alveolar ventilation in heterogeneously injured lung regions.

Finally, the temporal and integrative aspects of patient selection are likely to be decisive for the future of ECCO_2_R in ARDS. A strategy combining physiological metrics—such as compliance, ΔP, transpulmonary pressure, functional residual capacity (FRC) and dead-space fraction—with biomarkers reflecting inflammation, endothelial injury, and metabolic stress could enable more accurate identification of patients likely to benefit from extracorporeal CO_2_ removal. Such predictive enrichment approaches, integrating biological and mechanical phenotyping, may not only improve therapeutic precision but also enhance the interpretability and external validity of forthcoming clinical trials. As our understanding of ARDS pathobiology deepens, the targeted use of ECCO_2_R may thus evolve from a rescue technique into a rationally applied adjunct within a broader framework of personalized respiratory support. To make this paradigm more concrete, Figure 2 shows how different ARDS phenotypes may respond to ultra-protective ventilation when enabled by ECCO_2_R. By linking mechanical burden, inflammatory amplification, and regional strain to targeted physiological goals, the schematic outlines a phenotype-driven strategy rather than a one-size-fits-all intervention.

## 6. Technological and Practical Considerations

Ongoing technological refinements continue to enhance the safety, efficiency, and clinical integration of ECCO_2_R systems. Advances in hollow-fiber membrane composition, surface coating biocompatibility, and pump miniaturization have led to lower priming volumes and improved gas exchange efficiency at reduced extracorporeal blood flows [72].

Equally important are the innovations in blood pump design, which critically influence circuit performance and hemocompatibility. Blood pumps used in extracorporeal life support can be broadly divided into displacement pumps (e.g., roller or peristaltic pumps) and rotary pumps (e.g., centrifugal pumps) [72].

Peristaltic (roller) pumps move blood by cyclically compressing flexible polyvinyl chloride or silicone tubing with rollers. They provide a consistent flow independent of outlet pressure and prevent backflow, making them ideal for procedures requiring precise flow control, such as cardiopulmonary bypass [73]. However, shear stress–related hemolysis remains a concern, though modern designs have mitigated this risk [74].

Centrifugal pumps generate flow via a rotating impeller that produces a pressure gradient through centrifugal force. Flow depends on impeller speed and pressure differential, necessitating continuous monitoring [75]. The incorporation of magnetic levitation and hydrodynamic bearing systems has markedly reduced hemolysis and thrombosis, improving long-term reliability and safety [76,77]. Consequently, centrifugal pumps are now preferred for prolonged extracorporeal support due to their superior hemocompatibility and mechanical performance.

Moreover, the emergence of hybrid platforms capable of combining ECCO_2_R with renal replacement therapy (RRT) represents an important step toward multimodal organ support in critically ill patients [78,79,80]. In parallel, experimental approaches such as respiratory electrodialysis, the addition of carbonic anhydrase to the membrane or regional acidification of extracorporeal blood by means of an ion-exchange resin hold promise for achieving higher clearance rates with minimal circuit complexity [81,82,83].

Despite these innovations, anticoagulation remains a major determinant of safety and feasibility [11,53,84,85]. Systemic unfractionated heparin remains the standard approach, but alternative strategies—including regional citrate anticoagulation, heparin-bonded circuits, and optimized flow dynamics to reduce stasis—are under active investigation to mitigate bleeding and thrombotic complications [10,72,85,86]. Standardized training programs, simulation-based education, and rigorous adherence to operating protocols are essential prerequisites to ensure consistent and safe bedside implementation.

Economic implications remain uncertain. Although modeling studies suggest that ECCO_2_R could shorten the duration of invasive mechanical ventilation and intensive care unit stay—potentially offsetting its procedural costs—there is a lack of robust cost-effectiveness data [87]. Cost-effectiveness remains uncertain, with modeling studies suggesting potential reductions in duration of ventilation but with wide confidence intervals and high dependence on center experience.

## 7. Future Directions

**Figure 3:**
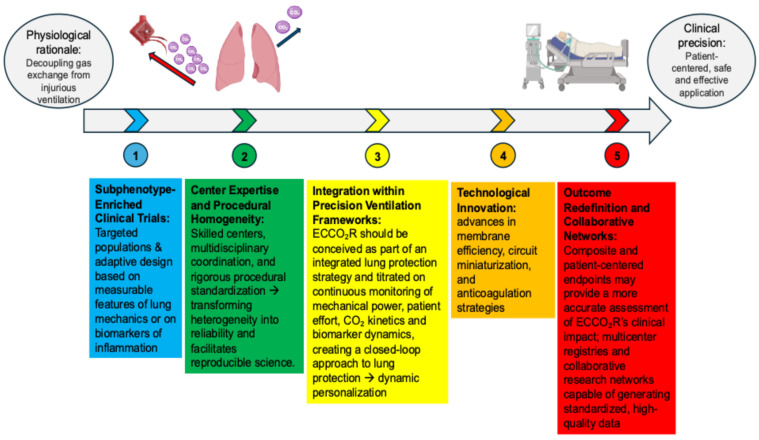
Conceptual framework summarizing the key priorities for designing future randomized trials to evaluate the clinical effectiveness of ECCO_2_R in ARDS.

The European Society of Intensive Care Medicine (ESICM) currently recommends against the routine use of ECCO_2_R for ARDS management outside randomized clinical trials [88]. Nevertheless, its physiological rationale remains compelling. The capacity to decouple gas exchange from injurious ventilatory patterns continues to embody one of the most intellectually attractive concepts in modern intensive care. Future advancement will depend on reconciling mechanistic insight with clinical precision—anchoring the technology within a patient-centered rather than population-based framework. To achieve this alignment, forthcoming randomized trials should address several key priorities (Figure 3):

- **Sub-phenotype-Enriched Clinical Trials**

Next-generation studies should move beyond unselected ARDS populations and focus instead on patients defined by specific physiological or biological signatures most likely to benefit from mechanical unloading. Adaptive designs, in which early physiological responses—such as a reduction in ΔP or respiratory effort—are used to adjust stratification or treatment allocation, may improve the ability to detect a true treatment effect. Enrichment strategies based on measurable features of lung mechanics (for example, compliance, ΔP, transpulmonary pressure, FRC or mechanical power) or on biological markers of inflammation could reveal benefits that remain hidden in more heterogeneous patient populations. In essence, ECCO_2_R should be tested where its physiological rationale most closely aligns with individual patient biology and clinical relevance.

- **Center Expertise and Procedural Homogeneity**

ECCO_2_R is a technically demanding intervention with a narrow therapeutic margin and a steep learning curve. Its safe and effective application requires the presence of robust extracorporeal expertise, multidisciplinary coordination, and rigorous procedural standardization. Restricting future trials to high-volume, experienced centers would enhance safety, minimize protocol variability, and ensure the interpretability of results. Concentrating research efforts in a limited number of expert hubs is not a constraint but a methodological necessity—one that transforms heterogeneity into reliability and facilitates reproducible science. The immediate goal is not widespread implementation but rigorous evaluation under optimal and controlled conditions.

- **Integration within Precision Ventilation Frameworks**

ECCO_2_R should be conceived as part of an integrated lung protective strategy, complementing prone positioning, individualized PEEP titration, and respiratory drive modulation. Continuous monitoring of mechanical power, patient effort, CO_2_ kinetics, and biomarker dynamics could enable titration of extracorporeal support intensity in real time, creating a closed-loop approach to lung protection. This integration would operationalize the concept of dynamic personalization, in which ventilatory and extracorporeal parameters evolve in response to the patient’s ongoing physiology rather than static protocol targets.

- **Technological Innovation**

Ongoing advances in membrane efficiency, circuit miniaturization, and anticoagulation strategies will be crucial to translating ECCO_2_R from conceptual promise to clinical practicality. Future systems should aim for reliable CO_2_ control, simplified operation, and seamless integration with existing ventilatory monitoring tools. The ultimate objective is to develop compact, semi-automated devices that can provide consistent extracorporeal support with minimal supervision. Such advances would enhance the practicality of ECCO_2_R and facilitate its evaluation and potential implementation within standard critical care practice.

- **Outcome Redefinition and Collaborative Networks**

Future research should move beyond mortality as the sole endpoint. Composite and patient-centered endpoints—such as ventilator-free days, recovery of lung function, health-related quality of life, and biomarker-defined resolution of inflammation—may provide a more accurate assessment of ECCO_2_R’s clinical impact. Given the complexity and relative rarity of this intervention, progress will depend on the establishment of multicenter registries and collaborative research networks capable of generating standardized, high-quality data. Integrating clinical, physiological, and biological information across centers will not only facilitate benchmarking and external validation but also foster collective learning and support the development of more targeted, hypothesis-driven trials in the future.

## 8. Conclusions

ECCO_2_R embodies a compelling physiological concept: extracorporeal CO_2_ removal enables ultra-protective ventilation, attenuating mechanical power and thereby reducing the risk of VILI in ARDS. However, despite its mechanistic elegance, technological maturation and renewed enthusiasm, conclusive clinical benefit has yet to be demonstrated. The heterogeneity of ARDS probably underlies these conflicting results and the gap between the physiological rationale and the outcomes. Future success will depend on rigorous phenotype-based trial design, concentration of expertise, and seamless integration into precision ventilation frameworks. Until such evidence emerges, ECCO_2_R should remain confined to experienced centers and controlled research environments.

Ultimately, as understanding of ARDS biology deepens, ECCO_2_R may evolve from a niche intervention to a rational, targeted component of advanced ventilatory care. Its success will ultimately depend on our ability to translate physiological insight into reproducible clinical benefit—turning an elegant concept into a precision therapy that truly protects the injured lung. ECCO_2_R should be re-framed not as a universal treatment for ARDS but as a targeted physiological tool integrated within precision ventilation strategies for biologically and mechanically selected patients. Future progress will depend on phenotype-enriched trials conducted in high-expertise centers and focused on clinically meaningful intermediate endpoints rather than mortality alone.

## Data Availability

No new data were created or analyzed in this study.

## Abbreviations

ARDS	Acute Respiratory Distress Syndrome
CO_2_	Carbon Dioxide
CRRT	Continuous Renal Replacement Therapy
ΔP	Driving Pressure
ECCO_2_R	Extracorporeal Carbon Dioxide Removal
ECMO	Extracorporeal Membrane Oxygenation
ESICM	European Society of Intensive Care Medicine
FiO2	Fraction of Inspired Oxygen
FRC	Functional Residual Capacity
H^+^	Hydrogen Ion
HCO_3_^−^	Bicarbonate Ion
IL-18	Interleukin-18
MeSH	Medical Subject Headings
PaCO_2_	Arterial Partial Pressure of Carbon Dioxide
PaO_2_	Arterial Partial Pressure of Oxygen
PAO_2_	Alveolar partial pressure of oxygen
PBW	Predicted Body Weight
PEEP	Positive End-Expiratory Pressure
pH	Potential of Hydrogen
P-SILI	Patient Self-Inflicted Lung Injury
RCT	Randomized Controlled Trial
RQ	Respiratory Quotient
RRT	Renal Replacement Therapy
VCO_2_	Carbon Dioxide Production
VILI	Ventilator-Induced Lung Injury
VO_2_	Oxygen Consumption
